# Tuning the Refractive Index Sensitivity of LSPR Transducers Based on Nanocomposite Thin Films Composed of Noble Metal Nanoparticles Dispersed in TiO_2_

**DOI:** 10.3390/ma16237355

**Published:** 2023-11-26

**Authors:** Diogo Costa, Marco S. Rodrigues, Eduardo Alves, Nuno P. Barradas, Joel Borges, Filipe Vaz

**Affiliations:** 1 Physics Center of Minho and Porto Universities (CF-UM-UP), University of Minho, Campus de Azurém, 4800-058 Guimarães, Portugalmarcopsr@gmail.com (M.S.R.); fvaz@fisica.uminho.pt (F.V.); 2 Instituto de Plasmas e Fusão Nuclear, Instituto Superior Técnico, Universidade de Lisboa, Av. Rovisco Pais 1, 1049-001 Lisboa, Portugal; ealves@ctn.tecnico.ulisboa.pt; 3 Centro de Ciências e Tecnologias Nucleares, Instituto Superior Técnico, Universidade de Lisboa EN10, 2695-066 Bobadela, Portugal; nunoni@ctn.tecnico.ulisboa.pt; 4 LaPMET—Laboratory of Physics for Materials and Emergent Technologies, University of Minho, 4710-057 Braga, Portugal; 5 Material Science Department, Transilvania University of Brasov, 29 Eroilor Blvd., 500036 Brasov, Romania

**Keywords:** reactive magnetron sputtered, thin films, noble metal nanoparticles, localized surface plasmon resonance (LSPR), plasmonic sensing, refractive index sensitivity

## Abstract

This work reports on the development of nanoplasmonic thin films consisting of Au, Ag, or Au-Ag nanoparticles dispersed in a TiO_2_ matrix and the optimization of the deposition parameters to tune their optical response. The thin films were produced by reactive DC magnetron sputtering of a Ti target with Au and/or Ag pellets placed on the erosion zone. The thicknesses (50 and 100 nm) of the films, the current density (75 and 100 A/m^2^) applied to the target (titanium), and the number of pellets placed on its surface were the deposition conditions that were used to tailor the optical (LSPR) response. The total noble metal content varied between 13 and 28 at.% for Au/TiO_2_ films, between 22 and 30 at.% for Ag/TiO_2_ films, and 8 to 29 at% for the Au-Ag/TiO_2_ systems with 1:1, 1:1.5, and 1:2 Au:Ag atomic ratios. After thermal annealing at 400 and 600 °C, LSPR bands were found for all films concerning the Au-TiO_2_ and Au-Ag/TiO_2_, while for Ag/TiO_2_, only for thin films with 28 and 30 at.% of Ag concentration. Refractive index sensitivity (RIS) was evaluated for Au and Au-Ag/TiO_2_ thin films. It was found that for bimetallic nanoparticles, the sensitivity can increase up to five times when compared to a monometallic nanoplasmonic system. Using Au-Ag/TiO2 thin films can decrease the cost of fabrication of LSPR transducers while improving their sensitivity.

## 1. Introduction

Optical biosensors are already widely implemented in the environmental, food, and medical industries [1,2,3] and comprise an optical transducer and a biorecognition element that mimics a biological interaction [4].

Localized Surface Plasmon Resonance (LSPR)-based optical sensors display promising results to be included in the next-gen of label-free detection methods [5,6,7,8] due to the numerous applications that can be derived from LSPR, such as sensing (both chemical [9,10] and biological [11,12,13,14]), phototherapies [15,16], optical imaging [17], catalysis [18,19], and surface-enhanced spectroscopies [20,21], among others [22,23,24].

LSPR is a widely studied optical phenomenon that occurs when the incident electromagnetic radiation interacts with NPs [25,26] with sizes one order of magnitude lower than the wavelength of the incident radiation [27,28,29]. This interaction results from the resonant oscillation of the conduction band electrons of the metals with the incident electromagnetic waves, leading to a strong extinction (absorption and scattering) band and an enhancement of the local electromagnetic field [25,30]. The LSPR band’s properties depend on the nanoparticles’ composition, concentration, size, shape, and distribution [31]. The result is that LSPR-based optical transducers are very sensitive to refractive index changes [32,33,34]. Therefore, even molecular interactions near the NP surface can be observed [35,36] without the use of label molecules [37], allowing for “label-free” detection using portable and low-cost systems with real-time analysis capabilities [38,39].

When the NPs are composed of noble metals, such as Au and/or Ag, the resonance conditions for the LSPR phenomena are met in the visible range of the electromagnetic spectrum [40,41,42]. Au and Ag are two of the most important and studied materials in the field of plasmonics. While Au NPs are biocompatible and mostly chemically inert [31,43,44], Ag NPs normally produce sharper LSPR bands compared to other metals due to their higher scattering efficiency [28,31,45]. However, Au and Ag are not limited to monometallic nanoparticles [15,46]. As both Au and Ag crystallize in face-centered cubic (fcc) structures, it is theoretically possible to obtain bimetallic Au-Ag NPs in a wide range of Au/Ag atomic ratios, allowing for further tuning of the LSPR position [47,48]. Recent studies relying on elemental mapping using Scanning Transmission Electron Microscopy with an Energy-Dispersive X-ray spectroscopy detector (STEM-EDX) have suggested the formation of bimetallic NPs composed of Au-Ag dispersed in a TiO_2_ dielectric matrix [49].

The study and development of LSPR-based optical transducers are performed using different approaches, one of which is the preparation of thin films composed of randomly dispersed NPs in a dielectric matrix. Compared to colloidal solutions of NPs, the solid supports keep the nanoparticles immobilized in the matrix, preventing nanoparticle aggregation, which can occur in colloidal systems [50]. Furthermore, thin films can achieve better signal-to-noise ratios (SNRs) and show higher reproducibility [51,52]. In these thin films, the NP growth follows well-established mechanisms, where three main stages are involved: (a) aggregation of the atoms until reaching the critical size and creation of a stable nucleus—nucleation phase; (b) growth of the stable nucleus at the expense of dissolved atoms in the matrix—“normal growth”; and (c) when the merge of two small NPs results in a larger NP—coalescence phenomenon, or when larger NPs are formed by the dissolution of smaller NPs by a large NP—Ostwald ripening [53,54].

To prepare the mentioned nanocomposite thin films, reactive DC magnetron sputtering can be used [55]. This technique allows for tuning of the structural and chemical characteristics of the thin films toward LSPR-based transducers [49,55,56]. In sputtering, the films’ properties can be changed by adjusting the preparation conditions, such as current density, noble metal content, target condition and material [56], deposition rate, and thickness [57]. However, to induce the NPs’ growth mechanisms, a second step of thermal annealing is necessary to obtain LSPR responses from these thin films [55,58], as the temperature during deposition normally does not rise above 200 °C, and, therefore, only small (bellow 10 nm) non-crystallized NPs are found after the deposition [59].

The objective of the present work was to study the influence of the deposition parameters on the refractive index sensitivity (RIS) of thin films composed of Au, Ag, or Au-Ag NPs dispersed in a TiO_2_ matrix. Reactive DC magnetron sputtering produced several samples of each plasmonic system with varying noble metal compositions and film thicknesses. The films were annealed at different temperatures to tune the LSPR response. Their transmittance spectra were studied, and experiments were conducted to determine the RIS of each sample, using media with different refractive indexes to observe LSPR band shift and choose the best candidates to be used as LSPR (bio)sensors working in transmittance mode (T-LSPR).

## 2. Materials and Methods

### 2.1. Nanoplasmonic Thin Film Production

Custom-made reactive DC magnetron sputtering equipment was used to prepare the nanoplasmonic thin films composed of Au, Ag, or Au-Ag NPs dispersed in a TiO_2_ dielectric matrix. The sputtering target was composed of titanium (99.99% purity) with a rectangular shape (200 × 100 × 6 mm^3^) and disks of noble metals (Au and/or Ag). For monometallic NP thin films (Au/TiO_2_ and Ag/TiO_2_), one or two disks of Au or Ag (16 and 32 mm^2^, respectively) were used. As for bimetallic NP thin films (Au-Ag/TiO_2_), combinations of half a disk of Au (8 mm^2^) and one quarter to one disk of Ag (4 to 16 mm^2^) were placed in the Ti target. The target was sputtered with a current density of 75 to 100 A/m^2^ (1.5 to 2.0 A) in a plasma composed of Ar (3.8 × 10^−1^ Pa) and O_2_ (3 × 10^−2^ Pa) for 9 to 22 min, to obtain thin films with thicknesses of approximately 50 and 100 nm. The working pressure was around 4.1 × 10^−1^ Pa, while the base pressure of the system was always below 6.0 × 10^−4^ Pa. The thin films were deposited onto polished fused silica (SiO_2_) for optical measurements and Si (p-type, Boron doped) for chemical characterization and were previously cleaned and activated with plasma. This was performed using a Zepto plasma system (Diener Electronic) using a 13.56 MHz generator, at a power of 50 W, first in an O_2_ atmosphere (60 Pa of pressure) for 5 min, and then in an Ar atmosphere (60 Pa of pressure) for 15 min. This activation step is usually used to remove contaminants from the substrate and to increase the adhesion of the thin films. Using these deposition conditions, sets of plasmonic thin films were produced with different thicknesses and noble metal compositions.

To induce the formation and growth of the NPs throughout the TiO_2_ matrix and achieve LSPR responses, the thin films were subjected to a post-deposition annealing treatment. This thermal treatment was performed in a vacuum furnace with a base pressure of approximately 10^−3^ Pa. The treatment protocol consists of a heating ramp of 5 °C/min until it reaches the desired temperature, an isothermal period of 5 or 1 h at 400 or 600 °C, respectively, and a final step of free cooling until it reaches room temperature.

### 2.2. Chemical Composition by Rutherford Backscattering Spectrometry

To determine the in-depth chemical composition of the produced thin films, Rutherford Backscattering Spectrometry (RBS) was used. The measurements were performed with a 2 MeV ^4^He^+^ beam, spotted in a 1 mm^2^ area, with an angle of incidence of 0° (normal incidence) and 25°. Three detectors were placed in the equipment chamber: a standard at 140° and two pin-diode detectors located symmetrically to each other at a 165° scattering angle. The RBS data were analyzed with the Ion Beam Analysis DataFurnace NDF v10.0a., and double scattering and pileup were calculated according to algorithms developed by Barradas et al. [60,61].

### 2.3. Optical Characterization of Nanoplasmonic Thin Films

Transmittance spectra, in the range of wavelengths from 300 to 900 nm, with a step of 0.5 nm, were measured using a UV-2450(PC) spectrophotometer from Shimadzu Corp. (Kyoto, Japan). The LSPR bands were analyzed using NANOPTICS software version 2022a [62] to obtain the LSPR peak position, namely, the wavelength of transmittance minimum (λmin) and transmittance minimum value (Tmin), which corresponds to the extinction maximum. It also allowed us to calculate the full width at half height (FWHH) and LSPR band full height (BFH), resulting from the difference between the Tmin in and the transmittance maximum at the band left-tail.

The sensitivity of the thin films produced was evaluated by measuring the optical response over time when their surface was immersed in different fluids with different refractive indexes. Considering a semi-infinite layer of surrounding media, the RIS calculation can be simplified to RIS = Δλ/Δƞ (nm/RIU), where Δλ is the LSPR band shift, and Δƞ is the difference between the refractive index of the used media [50,63]. For the measurements, a custom-made optical system from SARSPEC, LDA., composed of a white LED light source, a cuvette holder, and a CCD detector spectrometer, prepared to high resolution between 420 and 720 nm of wavelength, was used. Deionized water (η = 1.3325 RIU) and a 20% (*w*/*w*) sucrose solution (η = 1.3639 RIU) were used as media (for each half-cycle), and the LSPR band was monitored for 2 min per cycle. Spectra were acquired with a 3 ms integration time and an average of 200 scans. Using the NANOPTICS software, it was possible to process the acquired spectra and analyze the changes in the LSPR band [62] in detail.

## 3. Results

### 3.1. Chemical Analysis vs. Deposition Conditions

**Figure 1:**
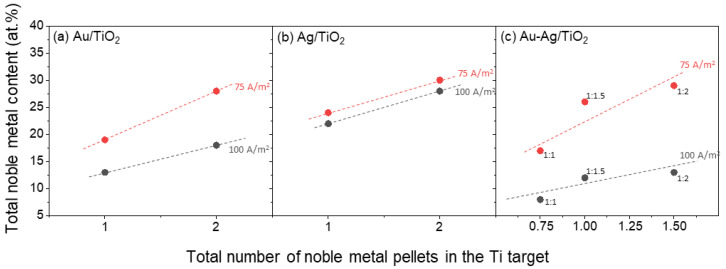
Total noble metal content measured by RBS for all thin film samples before annealing in function of the number of pellets used in the Ti target and the current density: (**a**) Au/TiO_2_; (**b**) Ag/TiO_2_; and (**c**) Au-Ag/TiO_2_.

Chemical characterization using RBS allows for quantifying the atomic concentration of the elements present in the thin films. In Figure 1, the total atomic content for each plasmonic system (as-deposited samples) is displayed as a function of deposition parameters (applied current and number of pellets placed in the target erosion zone).

Regarding Au/TiO_2_ thin films (Figure 1a), the Au content starts at 13 at.% and goes up to 28 at.%. The sample with 13 at.% of Au corresponds to the thin film prepared with one disk (pellet) of 16 mm^2^ of Au placed in the Ti target and a current density of 100 A/m^2^. As expected, increasing the Au amount in the Ti target, 16 mm^2^ to 32 mm^2^, without changing the applied current, led to an increase in noble metal content from 13 to 18 at.%. For the films produced with a current density of 75 A/m^2^, the Au content goes from 19 to 28 at.% for Au pellets’ area of 16 and 32 mm^2^, respectively. As has been previously shown by Rodrigues et al. (2018), decreasing the applied current from 2.0 A to 1.5 A leads to higher incorporation of noble metals in the film [56]. As such, for the same exposed area of Au (16 mm^2^), the sample produced with 100 A/m^2^ has a lower Au content (13 at.%) in comparison to the sample prepared with 75 A/m^2^ (19 at.%). Similar results were found for the samples prepared with an area of 32 mm^2^ of Au in the target, resulting in thin films with 18 and 28 at.% of Au in their composition, respectively. Furthermore, it is noticeable that there is a similar Au content for the samples produced with 32 mm^2^ of Au and 100 A/m^2^ of current density (18 at.%) and 16 mm^2^ and 75 A/m^2^ (19 at.%). However, it is expected that the morphology of the films differs, as the higher current (2.0 A) usually translates into denser thin films, while in the film prepared with 1.5 A, the columnar structure would be more easily observed [56].

Concerning the Ag/TiO_2_ thin films (Figure 1b), the samples present a higher noble metal content, from 22 to 30 at.%. This is due to the higher sputtering yield of Ag in comparison to Au [64,65]. Starting at one Ag disk in the Ti target (16 mm^2^) and 100 A/m^2^ of current density, the films have 22 at.% of Ag in their composition. Decreasing the current density from 100 to 75 A/m^2^ in Ag/TiO_2_ thin films increases the Ag content to 24 at.%, a similar trend as in the Au/TiO_2_ system. Incrementing the exposed Ag area in the Ti target from 16 to 32 mm^2^ led to an increase in Ag content in the thin films to 28 and 30 at.% for a current density of 100 and 75 A/m^2^, respectively.

Lastly, for Au-Ag/TiO_2_ (Figure 1c), a wider range of noble metal compositions was produced, and thus, the thin films were divided into three groups concerning the amount of Ag placed in the Ti target. Considering that the Au amount was fixed at half a disk (8 mm^2^) when preparing Au-Ag/TiO_2_ thin film with one-quarter of Ag disk (4 mm^2^), the ratio was found to be approximately 1:1 of Au to Ag, considering the error of the RBS analysis. At 100 A/m^2^, the total noble metal content is 8 at.% (4 at.% of Au plus 4 at.% of Ag). Decreasing the current density to 75 A/m^2^, the noble metal content doubled, with 9 at.% of Au and 8 at.% of Ag. Considering the thin films deposited with equal parts of Au and Ag in the Ti target (8 mm^2^ of each metal), the Au: Ag ratio increases to 1:1.5. With a current of 2.0 A, the Au composition was 5 at.%, while the Ag content increased to 7 at.%, with a total amount of noble metals of 12 at.%. Once again, decreasing the current to 1.5 A leads to an increase in total metal content. The Ag content increases to 16 at.%, while the Au stays roughly the same, at 10 at.%, when compared to the thin film with a 1:1 noble metal ratio. Finally, further increasing the exposed Ag area in the Ti target (to a full disk with 16 mm^2^) led to an Au: Ag ratio close to 1:2. The thin film prepared with 2.0 A contained 9 at.% of Ag, with Au content of 4 at.%, while at 75 A/m^2^, the film had an Au content of 10 at.%, and Ag content of 19 at.%. Considering the higher sputtering yield obtained for Ag, in comparison with Au, the chemical characterization results fall within expectations [64,65]. Previous studies with Au-Ag/TiO_2_ thin films, with a 1:2 ratio of Au to Ag [49], showed the formation of Au-Ag NPs with smaller Ag NPs dispersed in the TiO_2_ matrix. As such, for this study, we expect the same behavior for the 1:2 ratio, while for the 1:1 ratio, we expect mainly the formation of Au-Ag NPs. For the 1:1.5 ratio, an intermediate behavior is expected.

With this, the total amount of samples to analyze by optical characterization of the LSPR band is four different noble metal compositions for Au/TiO_2_ and Ag/TiO_2_ and six different compositions, two of each ratio, for Au-Ag/TiO_2_. Furthermore, each presented sample was produced with two different thicknesses, 50 and 100 nm, and while this deposition parameter does not affect the chemical composition, the optical response of the plasmonic thin films is influenced by this parameter.

### 3.2. Optical Characterization

#### 3.2.1. Au/TiO_2_

**Figure 2:**
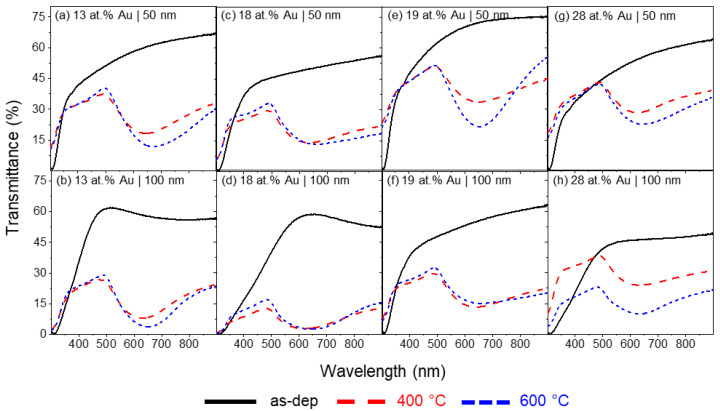
Optical transmittance spectra measured for Au/TiO_2_ thin films before and after annealing treatment at 400 °C and 600 °C.

The optical response and LSPR bands of the Au/TiO_2_ thin films before and after annealing treatment were evaluated using spectrophotometry in transmittance mode (Figure 2). Overall, all thin films as-deposited do not present LSPR bands, as expected, since after deposition, the Au aggregates have sizes below the quantum limit (<10 nm); quantum effects rule the optical properties [66], and, therefore, no LSPR band arises from these small NPs [59]. Instead, a typical interference-like behavior starts to appear for the thicker samples (100 nm) observed before the thermal treatments, as described in previous studies [56].After annealing, a drastic decrease in transmittance is observed for all annealed samples, both at 400 and 600 °C, revealing LSPR bands for all Au/TiO_2_ thin films. This is due to the growth of the Au NPs, overcoming the quantum size limit, and their crystallization throughout the TiO_2_ matrix. Additionally, the crystallization of the matrix in the anatase phase is also expected to occur since this crystalline structure usually starts to be formed at 300 °C [65], and the rutile phase occurs at higher temperatures > 600 °C [56]. The LSPR bands were analyzed using NANOPTICS software, and these parameters allowed us to characterize and compare the influence of the deposition and annealing conditions in the LSPR phenomena, as can be evidenced in previously published works [59]. A summary of the evaluated parameters for Au/TiO_2_ can be found in Table 1.

Starting with the sample with 13 at.% of Au, with 50 nm in thickness (Figure 2a), the LSPR band of the film annealed at 400 °C has its minimum positioned at λ_min_ = 642.4 nm and T_min_ = 18.0%, and the band has an FWHH of 246.0 nm and BFH of 19.6 pp (percentage points). Annealing this sample at 600 °C led to a redshift of the LSPR band minimum to λ_min_ = 665.1 nm, with a decrease in transmittance coordinates from T_min_ = 18.0% to T_min_ = 11.9%. This redshift can be associated with the increase in the size distribution of NPs and, thus, a higher scattering-to-absorption ratio [59]. Broadening of the LSPR band was observed, with an FWHH of 285.0 nm as well as an increase in BFH from 19.6 to 25.7 pp. Increasing the thickness of the thin film, from 50 to 100 nm led to an overall decrease in transmittance and a slight blueshift, accompanied by a narrowing of the LSPR band (Figure 2b). For this sample at 400 °C, the LSPR band has a λ_min_ = 62.8 nm for a T_min_ = 7.4%. Its band is also narrower, with an FWHH of 221.1 nm, while the BFH is similar to the thinner film, with 19.5 pp. Increasing the annealing temperature from 400 to 600 °C led to an expected redshift and decrease in transmittance, with the LSPR band positioned at λ_min_ = 639.8 nm and T_min_ = 3.3%, as well as broadening and increase in band height, 233.8 nm for FWHH and 25.7 pp for BFH.

Figure 2c depicts the LSPR band for the sample containing 18 at.% of Au, with 50 nm in thickness. This sample represents the increase in noble metal in the Ti target during deposition without changing the current density (100 A/m^2^). At 400 °C, the LSPR band is positioned at λ_min_ = 628.2 nm and T_min_ = 13.5%, which represents a blueshift and decrease in transmittance in comparison with the sample with 13 at.% of Au and 50 nm in thickness. In terms of band dimensions, the FWHH is 339.2 nm, and the BFH is 16.3 pp, representing a decrease in band height and a drastic broadening of the LSPR band. At 600 °C annealing, the expected redshift occurs from λ_min_ = 628.2 nm (for the same sample annealed at 400 °C) to λ_min_ = 642.1 nm, while the transmittance remained similar at T_min_ = 13.0%. There is an increase in BFH to 20.2 pp. However, a broadening of the LSPR band is observed, and the software could not calculate the FWHH of the spectra in the measured wavelength range due to the flattening of the LSPR band right-tail. Increasing the thickness of the sample with 18 at.% Au to 100 nm, the LSPR band suffered a blueshift (Figure 2d). For the sample annealed at 400 °C, the LSPR band minimum is located at λ_min_ = 616.0 nm and T_min_ = 2.3%. The LSPR band is considerably narrower, decreasing the FWHH from 339.2 nm (in the previous sample) to 217.6 nm, and with a smaller band height of 10.5 pp. Further annealing at 600 °C showed a redshift in the LSPR peak position, with the wavelength shifting to 627.1 nm, while the transmittance stayed similar, T_min_ = 2.1%, following the behavior of the sample with 18 at.% of Au and 50 nm in thickness. There is a broadening of the LSPR band, but, contrary to the previous sample, for the sample with 18 at.% of Au and 100 nm in thickness, the FWHH is measurable at 600 °C, increasing from 217.6 to 244.9 nm, with also an increase in BFH to 17.0 pp.

Figure 2e represents the LSPR bands for the samples composed of 19 at.% of Au and 50 nm in thickness. While the Au content is approximately the same as the previous sample, considering the RBS measurement error for Au, it differs in the applied current in the composite target (lowered from 2.0 A to 1.5 A), as well as the Au amount placed on it (one Au disk with 16 mm^2^). For this sample, at 400 °C, the LSPR peak minimum has a λ_min_ = 644.0 nm, representing a redshift in comparison to the sample with 18 at.% of Au and a T_min_ = 33.5%. In comparison, the LSPR band is narrower, with an FWHH of 274.9 nm, while the BFH is comparable at 17.8 pp. At 600 °C, a redshift occurs in the LSPR band, accompanied by a decrease in transmittance, following the tendency of the previous samples. The peak is located at λ_min_ = 648.7 nm and T_min_ = 21.5%. Moreover, the BFH almost doubles, increasing from 17.8 pp (at 400 °C) to 30.1 pp. However, contrary to the previous tendencies, the LSPR band suffers a narrowing after annealing at 600 °C, decreasing the FWHH to 208.0 nm. Regarding the thin film composed of 19 at.% of Au, but with 100 nm (Figure 2f), a blueshift of the LSPR band is observed, with the LSPR peak found at λ_min_ = 632.3 nm and T_min_ = 12.9% after annealing at 400 °C. The LSPR band is broad, with an FWHH of 295.4 nm, and its BFH is 16.9 pp, which is close to the sample with 19 at.% of Au annealed at 400 °C. After annealing at 600 °C, the LSPR band follows the redshift trend. The peak is located at λ_min_ = 637.5 nm and T_min_ = 13.3%. Similarly to the sample with 18 at.% Au, the annealing led to a broadening of the LSPR band, with the flattening of the band’s right tail, with no measurable FWHH and a BFH of 17.6 pp.

Finally, Figure 2g,h displays the LSPR bands’ spectra for the sample composed of 28 at.% of Au, prepared with two Au disks (32 mm^2^) in the erosion track of the Ti target and 75 A/m^2^ of current density, with 50 and 100 nm in thickness, respectively. For a thickness of 50 nm (Figure 2g), at 400 °C of annealing, the LSPR peak has the minimum positioned at λ_min_ = 621.6 nm and T_min_ = 27.9%. Compared to the sample with 19 at.% Au and similar thickness, it represents a blueshift in the LSPR band at this temperature and a decrease in overall transmittance, in line with the increase in noble metal atomic content. The LSPR band for this thin film is narrower, decreasing the FWHH from 274.9 nm (for Au/TiO_2_ with 19 at.% of Au and 50 nm in thickness) to 233.1 nm. At the same time, the BFH is slightly lower at 15.3 pp. Increasing the annealing temperature to 600 °C causes a redshift in the LSPR band, with the wavelength minimum shifting to λ_min_ = 673.2 nm and a transmittance decrease to T_min_ = 22.4%. A broadening of the LSPR band is also observed, increasing the FWHH from 233.1 to 273.3 nm while also increasing the BFH to 20.0 pp. Increasing the thickness to 100 nm at 400 °C results in a slight redshift, from λ_min_ = 621.6 to λ_min_ = 626.2 nm, with a decrease in transmittance, from T_min_ = 27.9% to T_min_ = 23.4%. A considerable widening of the LSPR band is also observable, with an FWHH of 344.4 nm due to the flattening of the right tail of the band. The BFH remains almost unaltered, with 15.3 pp to the 50 nm sample and 14.9 pp to the 100 nm thickness. Annealing this sample to 600 °C leads to a drastic decrease in transmittance, from T_min_ = 23.4 to T_min_ = 9.7%, with a 3 nm blueshift, from λ_min_ = 626.2 to λ_min_ = 623.2 nm. The increase in annealing temperature also reverted the band’s right tail flattening, narrowing the LSPR band significantly, decreasing the FWHH from 344.9 to 219.8 nm, and there was a slight decrease in approximately 1.5 pp in BFH, from 14.9 to 13.4 pp.

#### 3.2.2. Ag/TiO_2_

**Figure 3:**
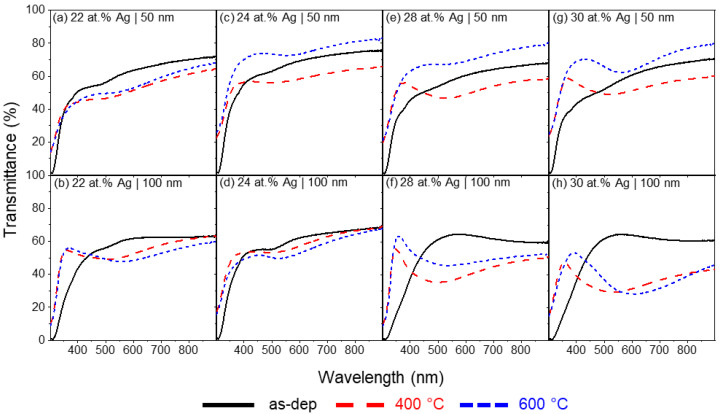
Transmittance spectra of Ag/TiO_2_ thin films as-deposited and annealed at 400 °C and 600 °C.

After preparing thin films with Au NPs dispersed in TiO_2_, thin films with dispersed Ag at different atomic percentages were also prepared by changing the number of Ag pellets in the erosion track of the Ti target and the applied current during the deposition. The transmittance spectra of Ag/TiO_2_ thin films were measured in all deposited films, as-deposited and annealed at 400 and 600 °C (Figure 3). A summary of the evaluated parameters for Ag/TiO_2_ can be found in Table 2.As observed in the Au/TiO_2_ thin films, no LSPR bands are observable in the as-deposited thin films. Furthermore, the thin films prepared with 22 at.% and 24 at.% of Ag for both 50 and 100 nm thick (Figure 3a–d) did not show LSPR bands after annealing either at 400 °C or 600 °C.

While the thin films prepared with 16 mm^2^ of Ag in the Ti target did not produce any well-defined LSPR band (Figure 3a–d), by analyzing the transmittance spectra of the thin films prepared with 32 mm^2^ of Ag, LSPR bands were observed (Figure 3e–h).

Figure 3e displays the transmittance spectra of the thin films composed of 28 at.% of Ag and 50 nm in thickness. Annealing at 400 °C produces an LSPR band with the minimum positioned at λ_min_ = 527.1 nm and T_min_ = 47.8%. The band FWHH is 213.8 nm, with a BFH of 9.6 pp. Increasing the annealing temperature to 600 °C led to an overall increase in transmittance and flattening of the LSPR band in such a way that the software could not unravel a band minimum. This behavior could also be observed in previous works, and it is related to the formation of fractal structures on the surface of Ag/TiO_2_ thin films [67,68]. Increasing the thickness for the thin films with 28 at.% of Ag (Figure 3f) causes the LSPR band at 400 °C to blueshift while also decreasing the transmittance. The LSPR peak is located at λ_min_ = 492.5 nm and T_min_ = 35.6%. Due to the higher BFH (20.1 pp) and the band’s flat right tail, the FWHH increases to 330 nm. Further annealing at 600 °C causes the LSPR band to drastically redshift to λ_min_ =577.1 nm and the transmittance to increase to T_min_ = 45.7%. The increase in annealing temperature also led to the flattening of the band’s right tail, causing NANOPTICS not to be able to measure the FWHH. The BFH decreased to 17.6 pp.

Decreasing the current density in the Ag-Ti composite target (with 32 mm^2^ of Ag) to 75 A/m^2^ increased the Ag atomic content to 30 at.%. With 50 nm thickness (Figure 3g) and annealed at 400 °C, the LSPR peak suffered a redshift when compared with the 50 nm thin film with 28 at.% of Ag. The transmittance minimum is located at λ_min_ = 521.4 nm and T_min_ = 49.2%. FWHH increased to 235.6 nm while the BFH remained almost unaltered at 9.1 pp. As seen in the sample with 28 at.% and 50 nm in thickness, after annealing at 600 °C, the transmittance of the LSPR band increased significantly. The band minimum is located at λ_min_ = 562.5 nm, which corresponds to a redshift when compared to the thin film annealed at 400 °C, and the transmittance increases to T_min_ = 63.4%. The FWHH decreases to 145.7 nm, as does the BFH to 6.9 pp. At this temperature, the Ag NPs normally have sizes above 100 nm [65] and diffuse for the film’s surface, decreasing the thin film thickness and leaving only smaller nanoparticles dispersed in the TiO_2_ matrix. Lastly, the transmittance spectra for the thin films composed of 30 at.% Ag and 100 nm thick are presented in Figure 3h. At 400 °C, the LSPR band minimum has λ_min_ = 532.8 nm and T_min_ = 29.5%. As such, the band has a redshift and lower transmittance when compared to the same composition for 50 nm. The FWHH of this band is 353.8 nm, and the BFH is 19.3 pp, revealing a much wider band when compared to the above-mentioned thin film. Annealing to 600 °C further increases the redshift in the LSPR band, with the minimum now located at λ_min_ = 604.8 nm and an almost unaffected transmittance of T_min_ = 28.1%. The band’s FWHH decreases slightly to 338.5 nm, and the BFH increases to 25.2 pp.

#### 3.2.3. Au-Ag/TiO_2_

To produce LSPR transducers that combine the best properties from Au and Ag nanoparticles, thin films with both Ag and Au dispersed in the TiO_2_ matrix were prepared as aforementioned. By changing the current density and the noble metal content in the Ti target, three groups of samples were produced, with ratios of 1:1, 1:1.5, and 1:2 of Au and Ag. As such, the optical response and LSPR bands of the thin films were also evaluated through spectrophotometry, and the data were analyzed using the software NANOPTICS. A summary of the evaluated parameters for Au-Ag/TiO_2_ can be found in Table 3.

**Figure 4:**
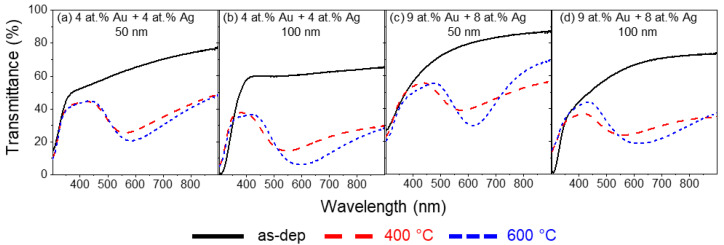
Transmittance spectra of Au-Ag/TiO_2_ thin films, with a composition ratio of approximately 1:1 of Au to Ag, as-deposited and annealed at 400 °C and 600 °C.

Starting with the thin films with approximately 1:1 ratio of Au to Ag (Figure 4), before annealing treatment, no LSPR bands were found in either of the analyzed samples since before annealing no nanoparticles could be found with sizes that contributed to the LSPR effect, as observed for Au/TiO_2_ and Ag/TiO_2_ thin films. After annealing at 400 and 600 °C, all thin film samples display LSPR bands in the transmittance spectra.

In Figure 4a, the transmittance spectra of the thin film with 4 at.% of Au and 4 at.% of Ag, deposited with a current density of 100 A/m^2^ and 50 nm thick, are displayed. After annealing at 400 °C, the LSPR band minimum is positioned at λ_min_ = 565.9 nm and T_min_ = 25.2%. The FWHH is 208.5 nm, and the BFH is 19.5 pp. Further annealing at 600 °C led to a redshift in the LSPR spectra, similar to Au/TiO_2_ thin films, and that could also be related to the increase in NPs’ size distribution, with the peak now positioned at λ_min_ = 583.6 nm and T_min_ = 20.4%. A slight broadening of the LSPR band is also observable, with an increase in FWHH to 217.9 nm, as well as an increase in BFH to 24.5 pp. Increasing the thickness of the thin film to 100 nm (Figure 4b) produces a blueshift at 400 °C, from λ_min_ = 565.9 to λ_min_ = 546.2 nm, for thicknesses of 50 nm and 100 nm, respectively. The overall transmittance spectrum decreases, while the transmittance minimum drops from T_min_ = 20.4 to T_min_ = 14.3%, which is an expected behavior since the thickness of thin films doubled. Moreover, such thickness increase to 100 nm also leads to a broadening of the LSPR band, with the FWHH increasing to 307.2 nm, while the BFH also increases, from 19.5 to 23.4 pp. Increasing the annealing temperature to 600 °C, decreases the overall transmittance, and causes a redshift of the LSPR band. The transmittance is reduced to T_min_ = 5.8%, and the band minimum is positioned at λ_min_ = 594.6 nm. Furthermore, there is a visible increase in BFH from 23.4 to 30.6 pp, while the FWHH remains almost unchanged, with a slight decrease from 307.2 to 300.9 nm.

As observed in the RBS analysis, decreasing the current density applied to the target increases the noble metal content in the thin films. In Figure 4c,d, the LSPR bands for thin films composed of 9 at% of Au and 8 at.% of Ag are shown. Starting with the thin film with 50 nm (Figure 4c) annealed at 400 °C, a redshift and an increase in transmittance are observed with the increase in noble metal content. The LSPR peak is located at λ_min_ = 575.8 nm and T_min_ = 38.8%. For this atomic composition, the LSPR band is marginally wider, with the FWHH increasing to 216.5 nm and the BFH decreasing to 16.9 pp. Still, in Figure 4c, the transmittance spectrum of this sample annealed at 600 °C reveals that increasing the annealing temperature leads to a redshift of the LSPR peak to λ_min_ = 617.2 nm and a decrease in the transmittance to T_min_ = 29.6%. Contrary to what might be expected, the LSPR band became narrower, with an FWHH of 148.3 nm and an increase in BFH to 26.1 pp. Finally, Figure 4d depicts the LSPR bands concerning the thin film with 9 at.% of Au and 8 at.% of Ag but with 100 nm in thickness. At 400 °C of annealing temperature and compared with the thin film with the same noble metal composition but with 50 nm in thickness, the LSPR peak blueshifted to λ_min_ = 560.9 nm, while the transmittance decreased to T_min_ = 23.7%. The LSPR band became wider, with an FWHH of 241.0 nm, due to the flattening of the band’s right tail, while the BFH slightly decreased to 13.2 pp. The same thin film annealed to 600 °C displays a redshift in its LSPR band, with λ_min_ = 613.4 nm and a decrease in transmittance to T_min_ = 18.5% compared to the same sample annealed at 400 °C. Although the band has a higher BFH, which increased from 13.2 to 25.5 pp, the band also became broader, with an FWHH of 310.6 nm, with a less flat band’s right tail.

**Figure 5:**
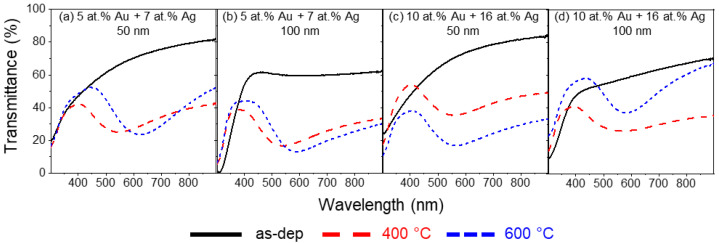
Transmittance spectra of Au-Ag/TiO_2_ thin films, with a composition ratio of approximately 1:1.5 of Au and Ag, as-deposited and annealed at 400 °C and 600 °C.

After the thin films with a 1:1 ratio, the Ag content placed in the erosion track of the Ti target was increased to 8 mm^2^, to a total of 16 mm^2^ of noble metals. This combination of Au and Ag results in a 1:1.5 ratio, and the different transmittance spectra are shown in Figure 5. Similar to the 1:1 ratio, no LSPR bands are present before the annealing treatment. After annealing at 400 and 600 °C, LSPR bands are present in all thin film samples.

When 100 A/m^2^ is applied to the composite target, the resulting composition is 5 at.% of Au and 7 at.% of Ag. The spectra corresponding to the thin film with 50 nm in thickness are displayed in Figure 5a. With the annealing temperature of 400 °C, the increase in Ag content results in a blueshift of the LSPR band when compared to the sample with 4 at.% of Au and Ag. The LSPR peak is located at λ_min_ = 545.5 nm and T_min_ = 24.9%. When compared to the sample of 1:1 ratio, the LSPR band is broader, with an FWHH of 229.7 nm and a lower BFH of 17.3 pp. Increasing the temperature to 600 °C leads to a redshift when compared to 400 °C. This shift could be related to the sublimation of Ag in excess, causing the LSPR band to have a higher contribution from Au. The band minimum is now located at λ_min_ = 622.3 nm and T_min_ = 23.3%. A slight widening is also observable, with the FWHH of 238.3 nm and an increase in BFH to 29.1 pp. Increasing the thickness to 100 nm (Figure 5b), with an annealing temperature of 400 °C, the LSPR band has the peak minimum located at λ_min_ = 539.6 nm and T_min_ = 16.6%, corresponding to a very slight band blueshift, compared to the thinner film. The band’s right tail has a mild flattening, which causes the FWHH to increase to 282.8 nm, while the BFH also increases slightly to 22.3 pp. After annealing at 600 °C, the band again suffers a redshift, with the peak positioned at λ_min_ = 592.96 nm of wavelength, and the transmittance decreases to T_min_ = 12.9%. The flat right tail causes the FWHH to increase drastically to 383.3 nm, with the BFH also increasing to 31.9 pp.

Changing the current density to 75 A/m^2^ in the deposition conditions reflects an increase in total noble metal content to almost double with an Au content of 10 at.% and 16 at.% of Ag (total noble metal content of 26 at.%). Analyzing the thin film with 50 nm in thickness (Figure 5c) annealed at 400 °C, the band presents a slight redshift, with the peak minimum found at λ_min_ = 554.9 nm, with an increase in transmittance to T_min_ = 35.4% when compared to a thin film with 5 at.% of Au and 7 at.% of Ag, with the same thickness and annealing temperature. Both thin films have similar BFH, 17.3 and 18.4 pp, for a total noble metal content of 12 and 26 at.%, respectively. The LSPR band became wider, with an FWHH of 271.8 nm, partially due to the flattening of the band’s right tail. Annealing the thin film at 600 °C causes the transmittance to decrease drastically, with the LSPR peak located at T_min_ = 16.9% of transmittance, while the wavelength minimum slightly red shifting to λ_min_ = 565.7 nm, compared to the thin film annealed at 400 °C. While the FWHH slightly increases to 282.2 nm, the right tail flattening seems to decrease, with the increase in BFH to 21.3 pp. Finally, increasing the thickness of the thin films with 10 at.% of Au and 16 at.% of Ag to 100 nm (annealed to 400 °C) leads to a decrease in transmittance to T_min_ = 26.0% and a redshift of the LSPR minimum wavelength to λ_min_ = 559.9 nm (Figure 5d). The LSPR band is broad and almost flat, with a larger FWHH (346.7 nm) and a reduced BFH (14.9 pp). Further annealing at 600 °C causes, unexpectedly, an overall increase in transmittance. The LSPR band has a redshift to λ_min_ = 585.1 nm and T_min_ = 37.0%. This increase in transmittance could also be related to the sublimation of excess Ag, decreasing the total noble metal content present in the thin film and increasing the overall transmittance. Moreover, the LSPR band is sharper, and the flattening of the right tail completely subsided, with an FWHH of 179.0 nm and a BFH of 21.3 pp.

**Figure 6:**
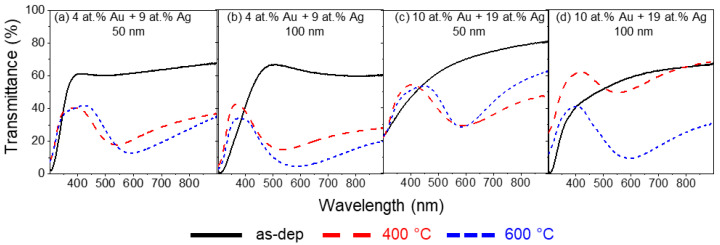
Transmittance spectra of Au-Ag/TiO_2_ thin films, with a composition ratio of approximately 1:2 of Au and Ag, as-deposited and annealed at 400 °C and 600 °C.

Lastly, the Ag content in the thin films was increased even further by placing 8 mm^2^ of Au and 16 mm^2^ of Ag in the Ti target. With this, the ratio of Au to Ag increased to 1:2, and their transmittance spectra are presented in Figure 6.

Starting with the thin film prepared with 100 A/m^2^ and 50 nm in thickness (Figure 6a), after annealing at 400 °C, the LSPR peak is located at λ_min_ = 540.6 nm, showing a blueshift of the LSPR band compared to the thin film of 1:15 ratio prepared in the same conditions, accompanied with a decrease in transmittance to T_min_ = 17.6%. Concerning the band’s shape, both FWHH and BFH increased slightly to 251.7 nm and 23.1 pp, respectively. At 600 °C, the band peak suffers the expected redshift from λ_min_ = 540.6 nm to λ_min_ = 589.0 nm and a decrease in transmittance to T_min_ = 12.6%. The band also became wider, with an FWHH of 297.3 nm and a higher BFH of 29.2 pp. Increasing the film thickness to 100 nm (Figure 6b) causes a visible decrease in overall transmittance when annealed at 400 °C, with the LSPR band peak with T_min_ = 14.7%, and slightly blueshifted to λ_min_ = 534.6 nm when considering the sample prepared with the same deposition conditions and 50 nm thick. Due to the high band flattening, which occurs at the right tail of the LSPR band, it was not possible to estimate a value of the FWHH for this sample, while the BFH increased to 27.8 pp. Increasing the annealing temperature to 600 °C promoted an overall decrease in transmittance, with the minimum positioned at T_min_ = 4.6% and a redshift, as expected, from λ_min_ = 534.6 to λ_min_ = 581.4 nm. The flattening of the band’s right tail subsided slightly, which led to a calculation of an FWHH 428.1 nm, which is the highest measured FWHH of all the thin films in this work. The BFH marginally increased to 29.9 pp.

Starting with the thin film prepared with 100 A/m^2^ and 50 nm thickness (Figure 6a), after annealing at 400 °C, the LSPR peak is located at λ_min_ = 540.6 nm, showing a blueshift of the LSPR band when compared to the thin film of 1:15 ratio prepared in the same conditions, accompanied with a decrease in transmittance to T_min_ = 17.6%. Concerning the band’s shape, both FWHH and BFH increased slightly to 251.7 nm and 23.1 pp, respectively. At 600 °C, the band peak suffers the expected redshift from λ_min_ = 540.6 nm to λ_min_ = 589.0 nm and a decrease in transmittance to T_min_ = 12.6%. The band also became wider, with an FWHH of 297.3 nm and a higher BFH of 29.2 pp. Increasing the film thickness to 100 nm (Figure 6b) causes a visible decrease in overall transmittance when annealed at 400 °C, with the LSPR band peak with T_min_ = 14.7% and slightly blueshifted to λ_min_ = 534.6 nm when considering the sample prepared with the same deposition conditions and 50 nm thick. Due to the high band flattening, which occurs at the right tail of the LSPR band, it was not possible to estimate a value for the FWHH of this sample, while the BFH increased to 27.8 pp. Increasing the annealing temperature to 600 °C promoted an overall decrease in transmittance, with the minimum positioned at T_min_ = 4.6% and a redshift, as expected, from λ_min_ = 534.6 to λ_min_ = 581.4 nm. The flattening of the band’s right tail subsided slightly, which led to a calculation of an FWHH 428.1 nm, which is the highest measured FWHH of all the thin films in this work. The BFH marginally increased to 29.9 pp.

The last set of Au-Ag/TiO_2_ thin films is comprised of atomic concentration ratios of 1:2 (Au to Ag), produced with a current density of 75 A/m^2^ (Figure 6c,d). For 50 nm thickness and 400 °C of annealing temperature, the LSPR band peak suffers a redshift to λ_min_ = 590.2 nm when compared to the thin film prepared with the current density of 100 A/m^2^ and a transmittance of T_min_ = 28.3%. The FWHH increases to 281.7 nm and a BFH of 25.0 pp. In the case of this thin film, increasing the annealing temperature to 600 °C causes no significant changes to the LSPR band peak position, with λ_min_ = 588.9 nm of wavelength and T_min_ = 28.4% of transmittance. However, the band became narrower, with an FWHH of 165.6 nm and a BFH of 25.7 pp. Lastly, increasing the thickness of the thin films composed of 10 at.% Au and 19 at.% Ag to 100 nm leads to a drastic increase in the transmittance spectrum. The transmittance increases to T_min_ = 49.4%. Compared to the sample with the same chemical composition and 50 nm, the LSPR band has a blueshift of the peak wavelength (λ_min_ = 552.6 nm). The band is considerably narrower, with an FWHH of 179.7 nm and approximately half of the BFH (21.9 pp). After annealing at 600 °C, the transmittance minimum drastically decreases to T_min_ = 9.5%., and the LSPR minimum has a wavelength position at λ_min_ = 594.6 nm. The band FWHH increases to 316.7 nm, corresponding to a widening of the LSPR band and an increase in BFH to 31.6 pp.

### 3.3. Refractive Index Sensitivity

To optimize the thin films with Au, Ag, or Au-Ag NPs dispersed in a TiO_2_ matrix as LSPR transducers, more than a simple evaluation of the LSPR band’s spectra must be performed. With this in consideration, the refractive index sensitivity was calculated by measuring the LSPR band of the thin films immersed in mediums with different refractive indexes. For this, cycles alternating deionized water (η = 1.3325 RIU) and a 20% (*w*/*w*) sucrose solution (η = 1.3639 RIU) were performed using a custom-made optical system from SARSPEC, LDA., with a modular spectrophotometer, where the detector is a CCD array, covering the wavelength range from 420 to 720 nm. This wavelength range reduction in the modular spectrometer was made to improve the resolution of the system. Nevertheless, it may exclude from the measurements all the samples that have wider bands, with large FWHH and bands with a significantly flat right tail. Despite that, such broader bands are not the most adequate for developing LSPR-based sensors.

**Figure 7:**
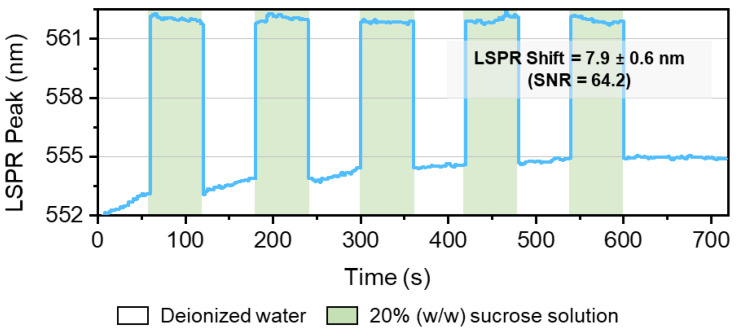
LSPR band minimum wavelength shift Au-Ag/TiO_2_ with 1:1 Au-Ag ratio, the total noble metal content of 17 at. % (9 at.% of Au and 8 at.% of Ag) and 50 nm in thickness, annealed at 400 °C. Analysis was performed in NANOPTICS software, showing the cycles due to the change in the refractive index of the surrounding media (deionized water vs. a model-solution of 20% (*w*/*w*) of sucrose). The last cycle is the reference cycle (water vs. water).

Therefore, the thin films manifesting the most adequate LSPR bands were analyzed using the above-mentioned method, and the resulting spectra were processed using NANOPTICS software (more details about the software can be found in [62]). An example of the resulting data for different cycles is depicted in Figure 7.

In Figure 7, the cycles due to the change in the refractive index of the surrounding media can be easily identified. The software fitted all the obtained spectra and calculated the LSPR peak position in each of them, returning the LSPR shift and standard deviation. It also determines the signal-to-noise ratio (SNR) and, by inputting the refractive index of each media, it estimates refractive index sensitivity (RIS).

The thin film in Figure 7 is composed of 9 at.% of Au and 8 at.% of Ag (1:1 ratio) and 50 nm in thickness, annealed at 400 °C; the LSPR shift is 7.9 ± 0.6 nm, with an SNR of 64.2. The RIS of this sample was estimated to be 250 ± 18 nm/RIU (refractive index unit), which was the sample manifesting the highest sensitivity of all the studied systems.

Table 4 and Table 5 present the results obtained for all evaluated thin films for Au/TiO_2_ and Au-Ag/TiO_2_, respectively.

Overall, the Au/TiO_2_ thin films have lower RIS for similar total noble metal content when compared to Au-Ag/TiO_2_ thin films. From all the analyzed Au/TiO_2_ thin films, most have an SNR below 10, which reveals that the measurement is highly affected by the noise, except the thin films with 13 and 28 at.% Au with 100 nm and annealed at 600 °C, and the thin film composed of 19 at.% Au with 50 nm in thickness, annealed at 400 °C. The higher RIS found for thin films with Au NPs is 77 ± 1 nm/RIU (an LSPR shift of 2.43 ± 0.04 nm and an SNR of 20.9), corresponding to the thin film with 28 at.% of Au, annealed at 600 °C. Note that the thin films with 18 and 19 at.% of Au have similar sensitivities (RIS), 59 ± 12 and 56 ± 1 nm/RIU, respectively. However, the thin film with 19 at.% has a much higher SNR, which could be related to the different microstructures developed during thin film production.

Concerning the Au-Ag/TiO_2_ nanoplasmonic system, only a few thin films do not have SNR higher than 10, namely, the thin film with 5 at.% Au and 7 at.% Ag (1:1.5 ratio) with 100 nm and annealed at 600 °C, the films with 10 at.% Au and 16 at.% Ag (1:1.5 ratio) with 100 nm, annealed at 400 or 600 °C, and the film with 10 at.% Au and 19 at.% Ag (1:2 ratio) with 100 nm, annealed at 600 °C. In the remaining analyzed Au-Ag/TiO_2_ thin films, the thin films annealed at 400 °C have a higher RIS than the ones annealed at 600 °C. This could be explained by the diffusion of Ag to the films’ surface and/or agglomeration of Ag in larger clusters after annealing at higher temperatures. It is also noticeable that within the same noble metal composition, the RIS is higher in thin films with lower thickness. One possible justification for this is the exposed portion of the nanoparticles being higher in the thinner films.

Noteworthy are the thin films with approximately the same total noble metal content, as in the case of the Au/TiO_2_ thin film with 19 at.% Au and the Au-Ag/TiO2 thin film with 9 at.% Au + 8 at.% Ag (total noble metal content of 17 at.%), and with the same thickness (50 nm) and annealed both at 400 °C. The bimetallic plasmonic system, with Au-Ag NPs, has an RIS almost five times higher than the monometallic plasmonic thin film. The results show the successful incorporation of Ag in the Au/TiO_2_ thin film system, with increased RIS, beneficial to the production of LSPR-based optical transducers for sensing applications.

## 4. Conclusions

This work reports on the optimization of deposition parameters using reactive DC magnetron sputtering to produce thin films composed of noble metals NPs (Au, Ag, or Au-Ag) dispersed in a TiO_2_ matrix toward the production of LSPR-based optical transducers. The thin films’ thicknesses used for this study were 50 and 100 nm, while the noble metal composition varied from 13 to 28 at.% for Au/TiO_2_ and 22 to 30 at.% for Ag/TiO_2_. For Au-Ag/TiO_2_, the thin films were divided by the noble metal ratio: 1:1,1:1.5, and 1:2 Au: Ag atomic ratio.

As expected, no LSPR bands were found before the annealing of the thin films. Annealing at 400 and 600 °C led to the development of LSPR bands in all nanoplasmonic systems by promoting the growth of the nanoparticles from the dispersed Au and Ag atoms in the TiO_2_. For Au/TiO_2_ thin film, generally, broad LSPR bands were found, with flatter bands’ right tails and the LSPR peak positioned above λ_min_ = 600 nm, due to the presence of only Au NPs. For Ag/TiO_2_ thin films, no LSPR bands were found for the thin films with Ag contents of 22 and 24 at.%. In the case of Au-Ag/TiO_2_, all thin films presented LSPR bands after annealing at 400 °C.

While no RIS measurement was possible for annealed Ag/TiO_2_ thin films, for Au nanoparticles dispersed in TiO_2_, the highest sensitivity was found with higher noble metal content, thickness, and annealing temperature. These thin films, composed of 28 at.% Au, 100 nm in thickness, and annealed at 600 °C resulted in an RIS of 77 ± 1 nm/RIU. Overall, for the bimetallic nanoplasmonic system, higher sensitivities were found for thin films with 50 nm in thickness and annealed at 400 °C. The highest sensitivity was measured for thin films composed of a noble metal ratio of 1:1 (9 at.% of Au and 8 at.% of Ag), with an RIS of 250 ± 12 nm/RIU. Furthermore, when considering similar total noble metal content, thickness, and annealing temperatures, the bimetallic nanoplasmonic system can be up to five times more sensitive than Au/TiO_2_.

In conclusion, the successful addition of Ag to the Au/TiO_2_ thin film can greatly improve the sensitivity of the LSPR transducer while decreasing its production cost, both by decreasing the total Au content used in the production and decreasing the annealing temperature needed to achieve a higher RIS.

## Figures and Tables

**Table 1:** Summary of LSPR band minimum position (λ_min_ and T_min_), FWHH, and BFH for Au/TiO_2_.nanoplasmonic thin films determined by NANOPTICS.

Noble Metal Composition	Thickness (nm)	Annealing Temp. (°C)	λ_min_ (nm)	T_min_ (%)	FWHH (nm)	BFH (pp)
13 at.% Au	50	400	642.4	18.0	246.0	19.6
		600	665.1	11.9	285.0	25.7
	100	400	625.8	7.4	221.1	19.5
		600	639.8	3.3	233.8	25.7
18 at.% Au	50	400	628.2	13.5	339.2	16.3
		600	642.1	13.0	-	20.2
	100	400	616.0	2.3	217.6	10.55
		600	627.1	2.1	244.9	17.0
19 at.% Au	50	400	644.0	33.5	274.9	17.8
		600	648.7	21.5	208.0	30.1
	100	400	632.3	12.9	295.4	16.9
		600	637.5	13.3	-	17.6
28 at.% Au	50	400	621.6	27.9	233.1	15.3
		600	673.2	22.4	273.3	20.0
	100	400	626.2	23.4	344.4	14.9
		600	623.2	9.7	219.8	13.4

**Table 2:** Summary of LSPR band minimum position (λ_min_ and T_min_), FWHH, and BFH for Ag/TiO_2_ nanoplasmonic thin films determined by NANOPTICS.

Noble Metal Composition	Thickness (nm)	Annealing Temp. (°C)	λ_min_ (nm)	T_min_ (%)	FWHH (nm)	BFH (pp)
28 at.% Ag	50	400	527.1	47.8	213.8	9.6
	100	400	492.5	35.6	330.0	20.1
		600	577.1	45.7	-	17.6
30 at.% Au	50	400	521.4	49.2	235.6	9.1
		600	562.5	63.4	145.7	6.9
	100	400	532.8	29.5	353.8	19.3
		600	604.8	28.1	338.5	25.2

**Table 3:** Summary of LSPR band minimum position (λ_min_ and T_min_), FWHH, and BFH for Au-Ag/TiO_2_ nanoplasmonic thin films determined by NANOPTICS.

Noble Metal Composition	Thickness (nm)	Annealing Temp. (°C)	λ_min_ (nm)	T_min_ (%)	FWHH (nm)	BFH (pp)
4 at.% Au + 4 at.% Ag	50	400	565.9	25.2	208.5	19.5
		600	583.6	20.4	217.9	24.5
	100	400	546.2	14.3	307.2	23.4
		600	594.6	5.8	300.9	30.6
9 at.% Au + 8 at.% Ag	50	400	575.8	38.8	216.5	16.9
		600	617.2	29.6	148.3	26.1
	100	400	560.9	23.7	241.0	13.2
		600	613.4	18.5	310.6	25.5
5 at.% Au + 7 at.% Ag	50	400	545.5	24.9	229.7	17.3
		600	622.3	23.3	238.3	29.1
	100	400	539.6	16.6	282.8	22.3
		600	593.0	12.9	383.3	31.9
10 at.% Au + 16 at.% Ag	50	400	554.9	35.4	271.8	18.4
		600	565.7	16.9	282.2	21.3
	100	400	559.9	26.0	346.7	14.9
		600	585.1	37.0	179	21.3
4 at.% Au + 9 at.% Ag	50	400	540.6	17.6	251.7	23.1
		600	589.0	12.6	297.3	29.2
	100	400	534.6	14.7	-	27.8
		600	581.4	4.6	428.1	29.9
10 at.% Au + 19 at.% Ag	50	400	590.2	28.3	281.7	25.0
		600	588.9	28.4	165.6	25.7
	100	400	552.6	49.4	179.7	21.9
		600	594.6	9.5	316.7	31.6

**Table 4:** LSPR band shift and RIS for Au/TiO_2_ nanoplasmonic thin films determined by NANOPTICS.

Noble Metal Composition	Thickness (nm)	Annealing Temp. (°C)	LSPR Shift (nm)	SNR	RIS (nm/RIU)
13 at.% Au	50	600	0.8 ± 0.2	1.5	25 ± 7
	100	400	0.5 ± 0.1	12.9	17 ± 3
18 at.% Au	50	400	0.9 ± 0.2	2.4	28 ± 5
	100	400	1.8 ± 0.4	1.7	59 ± 12
		600	0.8 ± 0.3	2.2	15 ± 2
19 at.% Au	50	400	1.75 ± 0.08	18.4	56 ± 1
28 at.% Au	50	400	0.8 ± 0.3	2.2	25 ± 10
		600	0.7 ± 0.4	1.3	22 ± 12
	100	400	0.4 ± 0.2	1.3	13 ± 5
		600	2.43 ± 0.04	20.9	77 ± 1

**Table 5:** LSPR band shift and RIS for Au-Ag/TiO_2_ nanoplasmonic thin films determined by NANOPTICS.

Noble Metal Composition	Thickness (nm)	Annealing Temp. (°C)	LSPR Shift (nm)	SNR	RIS (nm/RIU)
4 at.% Au + 4 at.% Ag	50	400	3.5 ± 0.1	71.8	110 ± 3
		600	1.0 ± 0.1	22.5	33 ± 2
	100	400	2.58 ± 0.03	30.2	82.2 ± 0.9
9 at.% Au + 8 at.% Ag	50	400	7.9 ± 0.6	64.2	250 ± 18
		600	0.51 ± 0.04	13.5	16 ± 1
	100	400	3.0 ± 0.3	28.3	96 ± 9
5 at.% Au + 7 at.% Ag	50	400	1.7 ± 0.1	14.8	53 ± 4
	100	400	1.1 ± 0.1	21.9	36 ± 2
		600	0.16 ± 0.05	3.4	5 ± 2
10 at.% Au + 16 at.% Ag	50	400	0.8 ± 0.2	6.6	22 ± 2
		600	0.9 ± 0.1	10.2	29 ± 4
4 at.% Au + 9 at.% Ag	50	400	0.8 ± 0.2	20.0	24 ± 7
10 at.% Au + 19 at.% Ag	50	400	5.8 ± 1.2	30.5	185 ± 35
		600	1.5 ± 0.1	52.6	49 ± 2
	100	600	0.4 ± 0.1	6.2	12 ± 3

## Data Availability

The data presented in this study are available on request from the corresponding author.
